# Supplementary material for: Association between oral microbiome and seven types of cancers in East Asian population: a two-sample Mendelian randomization analysis
Source: Front Mol Biosci. 2023 Nov 21;10:1327893. doi: 10.3389/fmolb.2023.1327893 (PMC10702768; doi:10.3389/fmolb.2023.1327893)
Supplement: Supplementary file 1 [file DataSheet1.ZIP › Supplementary Materials/MR plots of saliva/saliva═╝/ovarian cancer/pheno.676_to_ovarian cancer_forest.pdf]

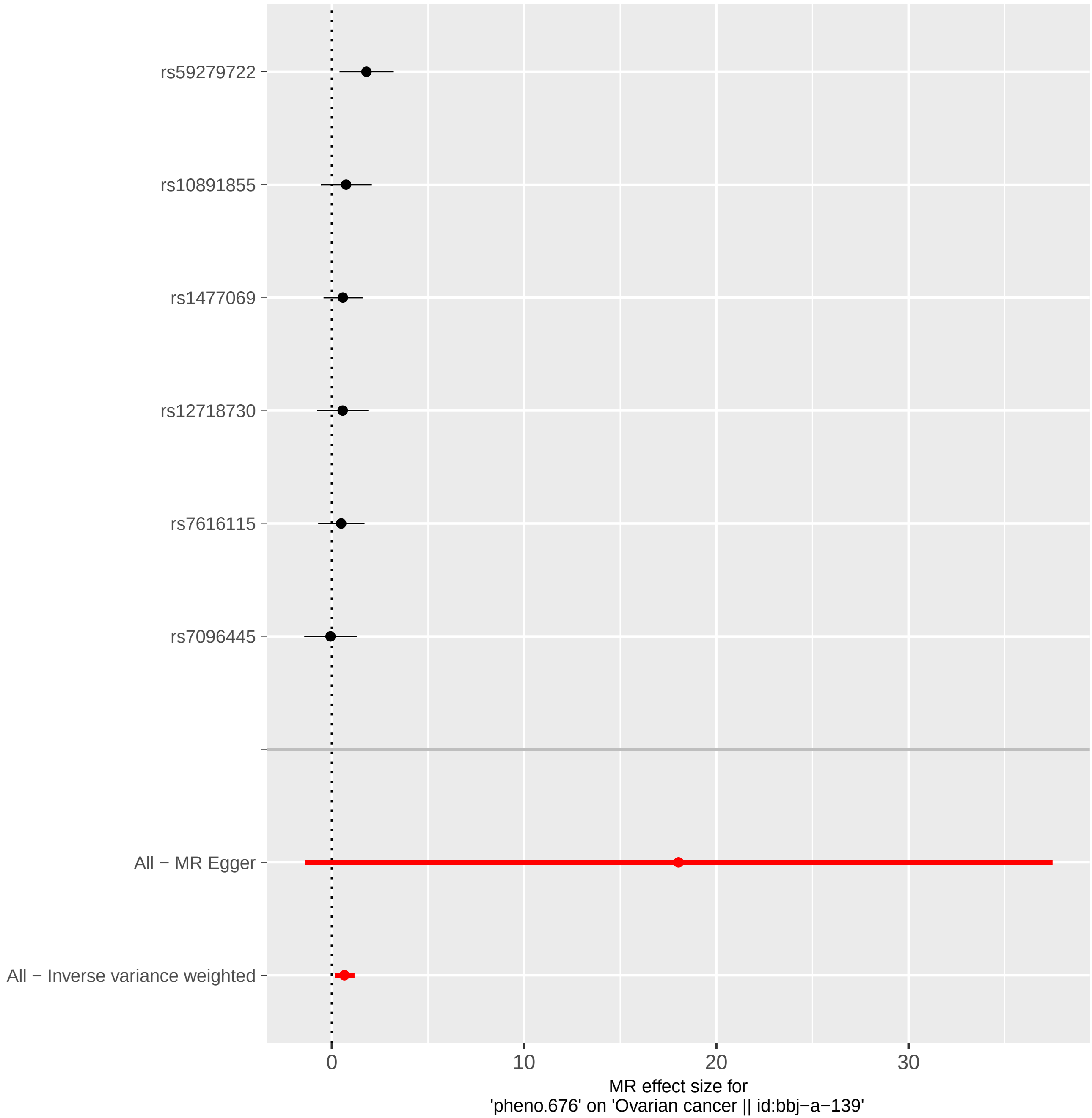
rs59279722
rs10891855
rs1477069
rs12718730
rs7616115
rs7096445
All − MR Egger
All − Inverse variance weighted
0
10
20
30
MR effect size for
'pheno.676' on 'Ovarian cancer || id:bbj−a−139'
